# Reducing Inappropriate Utilization of Albumin: The Value of Pharmacist-led Intervention Model

**Published:** 2018

**Authors:** Farzaneh Dastan, Hamidreza Jamaati, Habib Emami, Rodabeh Haghgoo, Raha Eskandari, Seyedeh Shadab Hashemifard, Fatemeh Khoddami, Zahra Mirshafiei Langari

**Affiliations:** a *Pharmacovigilance Department, National Research Institute of Tuberculosis and Lung Diseases (NRITLD), Shahid Beheshti University of Medical Sciences, Tehran, Iran.*; b *Clinical Pharmacy Department, School of Pharmacy, Shahid Beheshti University of Medical Sciences, Tehran, Iran.*; c *Chronic Respiratory Diseases Research Center (CRDRC), NRITLD, Shahid Beheshti University of Medical Sciences, Tehran, Iran. *; d *Tobacco Prevention and Control Research Center, National Research Institute of Tuberculosis and Lung Diseases (NRITLD), Shahid Beheshti University of Medical Sciences ,Tehran, Iran. *; e *School of Pharmacy, Shahid Beheshti University of Medical Sciences, Tehran, Iran.*

**Keywords:** Cost Saving, Clinical pharmacy, Drug utilization evaluation, Intervention study, Albumin, Inappropriate prescribing

## Abstract

Albumin is known as a human blood product, with high cost and limited availability. Several studies have demonstrated the extent in which albumin is being utilized in controversial indications not supported or weakly supported by the available literature. To rationalize the use of albumin and to decrease the inappropriate cost of this expensive drugˏ a two phase study, with equal length of 66-days, comprising an observational drug utilization evaluation and a pharmacist-led audit and feedback interventional study, was conducted in a tertiary referral hospital in Tehran, Iran. The results of the interventional phase including the introduction of evidence-base guideline for albumin via a pharmacist-led audit and feedback intervention was compared to the ones from the observational phase. A total of 90 and 45 patients were included in the phase one and phase two of the study respectively. During the initial phase, 1870 albumin vials were used, of which 1467 (78.4%) vials were prescribed inappropriately. Inappropriate use of albumin was decreased significantly by 79.3% (*p *< 0.001) through the interventional phase, leading to 38,800 USD reduction in inappropriate costs of albumin. Introduction of evidence based guideline in conjugation with pharmacist-led audit and feedback can significantly decrease the inappropriate use of albumin. These results also demonstrate shifting towards a more evidence-based practice, which can increase patient’s safety and enhance quality of care.

## Introduction

Human-Albumin (HA), a physiological plasma expander prepared from the plasma of healthy donor, is one of the most expensive non-blood plasma substitutes, which is mostly used to treat hypovolemia. According to a report in 2014, Iran is among the top 15 countries with highest per capita consumption of albumin (1). High cost of albumin and limited availability make it important to specify recommendations for its use. Crystalloids and non-protein colloids can be used as albumin alternative therapeutic strategies (2, 3).

Using albumin as the primary resuscitation fluid was shown to be beneficial in patients with severe sepsis or septic shock (4). Unfortunately, large meta-analysis failed to show consistency with these results (5, 6). On the other hand, albumin not only can be a harmful choice for the patient, it might also increase risk of mortality (4, 7). As an example, albumin can precipitate edematous status like pulmonary edema if used in critically ill patients with increased microvascular permeability; with trauma or burns (8, 9). Furthermore, administration of albumin to resuscitate patients with traumatic brain injuries was linked with an increased risk of mortality (10). Even today a great percentage of albumin is being used inappropriately which therefore has a negative impact on total pharmaceutical expenditure. Despite the existence of proven data against its actual benefit, albumin is still being used as a nutritional intervention (10). Furthermore, SAFE study, comparing albumin to normal saline for fluid resuscitation in critically ill patient, demonstrated no significant difference between the two groups (4).

Previous drug utilization evaluation (DUE) study performed on albumin in a tertiary hospital in Iran showed 36.2% inappropriate use of albumin (11). The primary objective of this study was to rationalize the use of albumin in the hospital, by activating a pharmacist driven process for daily orders of albumin over a period of 66 days.

*Methods*

*Initial DUE study*

In an effort to rationalize and reduce the inappropriate cost of HA use, we have conducted a pharmacist interventional study in National Research Institute of Tuberculosis and Lung Disease (NRITLD), which is one of the affiliated teaching hospitals by ShahidBeheshti Medical University. The initial DUE study was undertaken between 13^th^ February 2016 and 18^th^ April 2016 and approximately comprised 70 percent of the patients, taking albumin in this time frame. The patients were randomly chosen from 15 out of the total 20 medical wards; a form designed and filled for each patient on daily bases by a Doctor of pharmacy student. To ensure consistency in interpretation of the study criteria, a clinical pharmacist examined data collected by the Doctor of pharmacy Student. 

*Extension Interventional Study*

The extension study was taken place between 2nd April 2016 and 29^th^June 2016 in NRITLD, a center with 446 beds and 20 medical wards. All adult inpatients at the hospital receiving albumin beyond 3 consecutive days and during the 66-day period were included in the extension descriptive prospective study. The previously designed form was made available through the hospital network common space for all medical wards.

In order to monitor the appropriateness of albumin use and to examine the physician’s compliance with the protocol, the pharmacy activated an ordering process for daily requests of albumin. Each hospital unit sent a daily request for medication to pharmacy through a computerized system (HIS). An automatic system lock was set on albumin if it was ordered for more than 3 consecutive days. Consequently, physicians were required to fill out the albumin request form and send the form to pharmaceutical care department to be reviewed by clinical pharmacist. The forms were submitted daily over a period of 66 days. 

Criteria for appropriate use were developed from studies literally available in hand and guidelines derived from expert consensus (12,13). These criteria were then reviewed and endorsed by Drugs and Therapeutics Committee. Data collected included demographic information; a list of appropriate and inappropriate indications; quantity of albumin administered; serum albumin, total protein, serum creatinin and patient’s status. Moreover, it was also possible for the physician to indicate «out of guideline request» if none of the indications listed was suitable. In this case however, the physician was asked to present documents to support “out of the guideline” indication of use. 

*Statistical Analysis*

Analysis of the data was based on data frequencies and data summaries (mean, patient count). We have also summarized our data using Chi-Square Test which was applied for nominal data; Independent T-test and Mann-Whitney U Test were employed for analyzing the parametric and non-parametric data, respectively. *p*-value < 0.05 was considered statistically significant.

Data were collected and analyzed using IBM SPSS Software version 22.0 (SPSS, Inc., Chicago, IL, USA). 

## Results

*Initial DUE Study*

Ninety patients received a total of 1870 albumin vials during the 66-day study period. There were 49 men and 41 women, with a mean age of 58.48 ± 20.40 (± Standard deviation) years. Generally, 1467 (78.4%) of the albumin vials were prescribed inappropriately, and this comprised 77 cases included in the initial study. Inappropriate cost of albumin use was 48,900 USD, with an estimated annual cost of 266,460 USD (Table 1).

Specialty with more tendency to prescribe albumin was critical care medicine, prescribing 725 (38.8%) of the ordered vials. Pulmonary medicine specialty was the second (394 Vials, 21%) and oncology prescribed the least number of times (7 Vials, 0.4%). 

Only in 13 cases (14%), albumin was prescribed in accordance to the guideline. Moreover, in half of the included cases, during phase I study, albumin was prescribed appropriately in the beginning, but in 30 (33%) cases, its continuation was inappropriate beyond 3 days. Critical care medicine had the highest percentage (18 Cases, 20%) of appropriately prescribed cases in day one; however, extension of therapy beyond 3 days with an inappropriate indication (23 Cases, 26%) made continuation of therapy inappropriate. Surgery ward prescribed albumin 15 times, which all 15 cases were inappropriate uses of albumin (Table 2).

Prescriptions were most frequent when albumin level was above 2.5, making it the most common inappropriate indication of use. Continuing albumin beyond 3 days inappropriately was the second most improper indication for albumin use. The most frequent appropriate Indication was serum albumin below 2.5 (10, 11.1%) (Table 3).

*Extension interventional study*

After implementation of albumin guideline, in a 66-days period, 45 requests were made, of which 487 vials (61.6%) were prescribed appropriately. The reduction in unjustified use of albumin was 79.3% (1467 vs. 303), and was found to be statistically significant (*p *< 0.001), leading to estimated annual cost saving of 211,600 USD. The reduction in inappropriate use of albumin was 93%, 92%, 86% and 78.5% in pulmonology, surgery, nephrology and critical care respectively (*p* < 0.001) (Table 1). Since our hospital has no gastroenterology ward, our data did not account for indications that albumin is being utilized for hepatic and gastrointestinal indications.

There were 35 men and 10 women, with average age of 52.5 ± 17.47 (± SD) years. The majority of albumin used were deemed to have been inappropriate, most often due to failing to meet the criteria, namely because patients had serum albumin above 2.5 mg/dL (Table 3).

Reduction in mean number of albumin vials used per patient (*p* = 0.235) and length of treatment (*p* = 0.153) were observed comparing to the previous study, but this reduction was not significant. (Table 4).

## Discussion

Albumin, to a great extent, is utilized in indications that are weakly supported by the available literature, or for indications which are not supported by current evidence (13,14). Use of albumin was shown to be mostly inappropriate, especially results of drug utilization evaluation studies in Iran, demonstrated, an extremely high unjustified utilization of albumin (7,15,16). Albumin DUE, done by Farsad *et al* (17), showed 93.7% inappropriate use of drug in a cardiovascular, medical, and research center in Tehran, Iran. Similar results were reported by Kazemi *et al. *(18). Inappropriate use of albumin in our hospital has risen dramatically, from 36.2% to 78.4% since the previous DUE, which took place in 2012 (11). We deliberately focused on rationalization of albumin use, and possibly decreasing hospital unnecessary expenses.

Introduction of evidence-base guideline for albumin, via a pharmacist-led intervention, resulted in 79.3% (*p *< 0.001) reduction in inappropriate use of albumin. The intervention was done by introducing a model, through generation of evidence based guideline, computer decision support program, implementation of stop orders in Hospital Information System (HIS), and audit and feedback based on consulting physicians via telephone calls. Several studies have highlighted how audit and feedback can be effective in rationalizing physician ordering patterns (19,20). A review done by Jamtvedt *et al,* concluded that “Audit and feedback can be effective in improving professional practice. The relative effectiveness of audit and feedback is likely to be greater when baseline adherence to recommended practice is low and when feedback is delivered more intensively” (20).

Many studies and systematic reviews have shown the positive impact of pharmacist-led interventions, on patient’s safety, and healthcare cost reduction (21, 22). One study done by Nazari *et al*. have suggested that clinical pharmacists have an important role in educating nursing staff; They decreased drug-food interactions significantly after nurses have attended lectures delivered by clinical pharmacists (23). Furthermore, Avery *et al*. demonstrated the cost effectiveness of adding a Pharmacist-led information technology complex intervention (PINCER) to simple feedback for medication error (24). Another study showed that medication errors were significantly decreased through clinical pharmacist intervention in orthopedic and gastroenterology wards (25).

 In our interventional study inappropriate use of albumin was decreased by 79.3%, demonstrating shifting toward a more evidence based practice in our hospital. Few similar studies have attempted to rationalize the use of albumin through different modalities. Mahmoudi *et al*. decreased use of albumin by 36% in a university Hospital in Shiraz, Iran (26). Another study done in University of Michigan Hospital, demonstrated a 10% reduction in total quantity of albumin used and the author concluded that targeted educational intervention can reduce inappropriate albumin use and thereby controlling rising healthcare cost (27). Furthermore, a research published in British Colombia Medical Journal in 2012, studied the effect of introduction of guideline on rationalization of albumin utilization. Appropriate indications were raised from 22% to 56%, however the author accounts this results discouraging since 39.4% of orders are still inappropriate (28).

Results of our study shows a dramatic rise in appropriate use of albumin from 21.6% to 61.6%, by clinical pharmacist intervention which can lead to estimated annual cost saving of 211,600 USD; nevertheless albumin is still being utilized inappropriately in 38.3% of the cases. This highlights the importance of using other interventional means to further decrease the inappropriate use of albumin, in the future. As a conclusion, using targeted physician interventions is recommended by the authors to raise compliance of the prescriber to hospital guidelines.
